# Ultrasound-induced Cavitation enhances the efficacy of Chemotherapy in a 3D Model of Pancreatic Ductal Adenocarcinoma with its microenvironment

**DOI:** 10.1038/s41598-019-55388-0

**Published:** 2019-12-12

**Authors:** R. Leenhardt, M. Camus, J. L. Mestas, M. Jeljeli, E. Abou Ali, S. Chouzenoux, B. Bordacahar, C. Nicco, F. Batteux, C. Lafon, F. Prat

**Affiliations:** 1 University of Paris Descartes, INSERM U1016, Cochin Institute, Paris, France; 2 Sorbonne University, APHP, Saint-Antoine Hospital, Paris, France; 3 LabTAU, INSERM U1032, Centre Léon Bérard, Université-Lyon 1, Lyon, 69003 Lyon, France

**Keywords:** Gastroenterology, Oncology, Gastrointestinal cancer

## Abstract

Pancreatic ductal adenocarcinoma (PDAC) is supported by a complex microenvironment whose physical contribution to chemoresistance could be overcome by ultrasound (US) therapy. This study aims to investigate the ability of US-induced inertial cavitation in association with chemotherapy to alter tumor cell viability via microenvironment disruption. For this purpose, we used a 3D-coculture PDAC model partially mimicking the tumor and its microenvironment. Coculture spheroids combining DT66066 cells isolated from KPC-transgenic mice and murine embryonic fibroblasts (iMEF) were obtained by using a magnetic nanoshuttle method. Spheroids were exposed to US with incremental inertial cavitation indexes. Conditions studied included control, gemcitabine, US-cavitation and US-cavitation + gemcitabine. Spheroid viability was assessed by the reduction of resazurin and flow cytometry. The 3D-coculture spheroid model incorporated activated fibroblasts and produced type 1-collagen, thus providing a partial miniature representation of tumors with their microenvironment. Main findings were: (a) Gemcitabine (5 μM) was significantly less cytotoxic in the presence of KPC/iMEFs spheroids compared with KPC (fibroblast-free) spheroids; (b) US-induced inertial cavitation combined with Gemcitabine significantly decreased spheroid viability compared to Gemcitabine alone; (c) both cavitation and chemotherapy affected KPC cell viability but not that of fibroblasts, confirming the protective role of the latter vis-à-vis tumor cells. Gemcitabine toxicity is enhanced when cocultured spheroids of KPC and iMEF are exposed to US-cavitation. Although the model used is only a partial representation of PDAC, this experience supports the hypothesis that US-inertial cavitation can enhance drug penetration and cytotoxicity by disrupting PDAC microenvironment.

## Introduction

Pancreatic ductal adenocarcinoma (PDAC) is one of the most dreadful malignancies with a high mortality rate and poor prognosis. PDAC’s incidence increases rapidly and is expected to be the second leading cause of cancer death in the Western world within the upcoming decade^1^, whereas the overall 5-year survival rate of less than 5% has not significantly improved recently. Current treatment for PDAC includes aggressive chemotherapy and surgical resection, but only 20% of patients are deemed suitable for attempted curative resection because of the early local and metastatic spread of the disease^2^. Chemotherapy is the standard-of-care for locally advanced and metastatic cases^3^ but evidence has recently accumulated to consider the tumor microenvironment as the mainstay of chemoresistance in PDAC patients. Pancreatic stellate cells (PSCs), also known as carcinoma-associated fibroblasts (CAFs) are among the most important host cells of the microenvironment. They are largely involved in the strong desmoplastic reaction almost constantly observed in PDAC^4^ and thus play a significant role in chemoresistance, since chemotherapeutics agents, although highly efficient in PDAC cells *in vitro*, are either sequestered within or not allowed to penetrate into the stroma and reach malignant cells^5^. Chemocytotoxicity is also hampered by hypoxia and hypovascularization in the tumor microenvironment whereas immune checkpoint inhibitors cannot be targeted efficiently probably in part for the same reasons. Therefore, the obvious and urgent need to develop innovative treatments in PDAC suggests that rather than more efficient anticancer drugs, stromal disruption or remodeling is the most promising way to improve PDAC prognosis. One method to achieve that goal is by using physical agents with a capacity to dramatically modify stromal properties such as its internal cohesion forces and elasticity, interstitial pressure, oxygenation and so on. The multicellular tumor spheroid model has emerged as a powerful tool for pathophysiology and anti-tumor research^6^. Three-dimensional tumor spheroids have enabled more accurate characterization of tumor cell behaviors by obtaining a 3D architecture^7–9^. These culture models are useful and relevant to evaluate new therapeutic methods.

Therapeutic, high intensity ultrasound (US) used for drug and gene delivery^10^ is one such method. US-induced cavitation is a non-thermal, mechanical phenomenon whose “inertial” or transient form may be generated to increase the therapeutic effect of chemotherapy by promoting the uptake of molecules via the permeation of cell membrane and/or by inducing microenvironment disruption^11^. Our research group previously reported the efficacy of ultrasonic inertial cavitation to induce a reduction of tumor growth in a monocellular spheroid model of PDAC^12^. In a further step of this research, we wanted to determine whether cavitation has the capacity to alter tumor viability and chemoresistance when tumor cells are supported by some key elements of the stroma. For this purpose, we developed a 3D model composed of both tumor cells and activated fibroblasts mimicking pancreatic cancer with its microenvironment.

## Results

### Characterization of coculture spheroids

**Figure 1:**
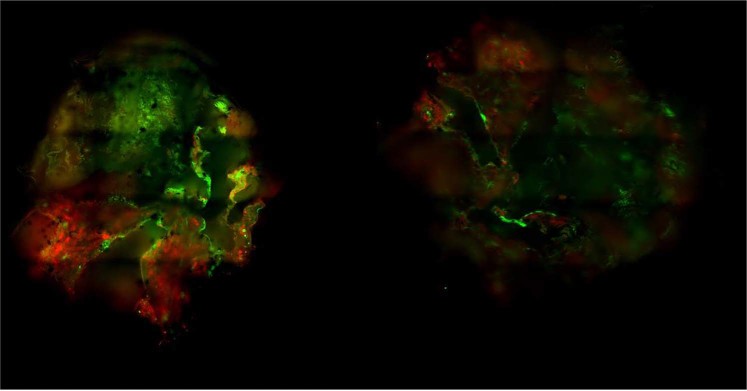
Immuno-fluorescence imaging of KPCF spheroid at day 3 of growth. KPC and iMEFs respectively expressed mCherry (red) and Green Fluorescent Protein (GFP) for clear immuno-fluorescent demarcation of both components.

Immuno-fluorescence imaging at day 3 of growth of KPC murine adenocarcinoma + murine embryonic fibroblasts (iMEF) cocultured spheroids (KPCF spheroids) showed the co-localization of m-cherry and GFP expression in, respectively KPC and fibroblast cells. The architectural organization of the spheroid was heterogeneous according to the different sections observed. Figure 1 illustrates the random distribution of both cell types within the spheroid. There was no pattern of intra-spheroidal regionalization between tumor cells and fibroblasts. 3D cocultured tumor spheroids reached a mean area of 300 µm (SD = 2.4) at their broadest cross-section within 3 days of culture.

**Figure 2:**
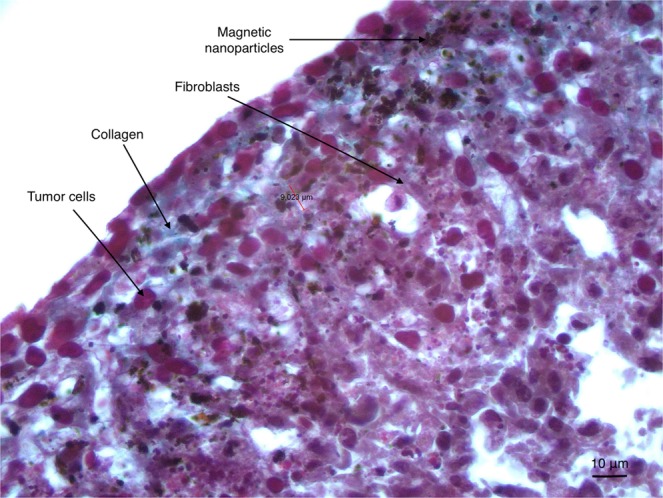
Histological section (5 microns) of a KPCF spheroid after 10 days of incubation. Staining with HE (Hematoxylin Eosin) and Masson’s Trichrome. Collagen fibers appear in blue. Blackish deposits correspond to residual magnetic nanoparticles.

After a standard staining by HE and Masson’s Trichrome, histological analysis at day 10 (*ie* after nanoparticle clearance allowing paraffin embedding and section) demonstrated the presence of collagen synthesized by fibroblasts, visible in blue. The periphery of the spheroid was characterized by a connective tissue displaying an arrangement similar to that of an epithelial layer. Although the center of the spheroid was composed of a majority of dead cells after 10 days of growth, both types of cells remained clearly distinguishable in the spheroid periphery. This histological section presented in Fig. 2 illustrates the similarities between a human PDAC tumor section and the coculture spheroidal model with its necrotic center, the existence a cell viability gradient increasing from the center to the periphery, the intricate arrangement of cancer cells and activated fibroblasts producing collagen, forming the basis of a tumor microenvironment.

### Comparison of gemcitabine cytotoxicity in cocultured spheroids *vs* monotypic KPC spheroids

**Figure 3:**
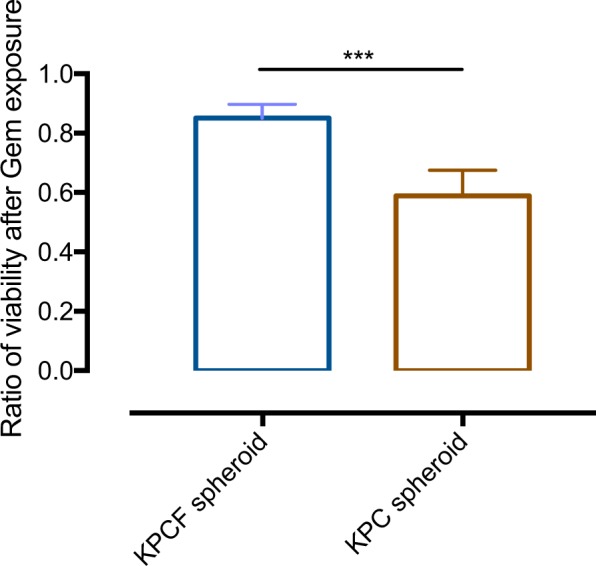
Ratio of viability between KPCF and KPC spheroids treated with Gem compared to control. Viability was measured 24 h after low-dose (5 μM) Gemcitabine incubation: Comparison of KPCF (KPC + fibroblasts) *vs* KPC (monotypic) spheroid models (n = 6 spheroids for each condition).

Gemcitabine decreased viability in both cocultured and monotypic KPC spheroid models (Fig. 3). Low-dose Gemcitabine cytotoxicity after 24 h of incubation was significantly reduced in the presence of KPC + iMEFs compared with KPC cells alone. As both groups included different numbers of cells, the analysis evaluated the ratio of viability between Gem group and their own control group. The ratio of viability after Gem treatment was significantly superior in KPCF than in KPC spheroids (p < 0.01).

### KPCF spheroid viability after ultrasonic cavitation combined to Gemcitabine

**Figure 4:**
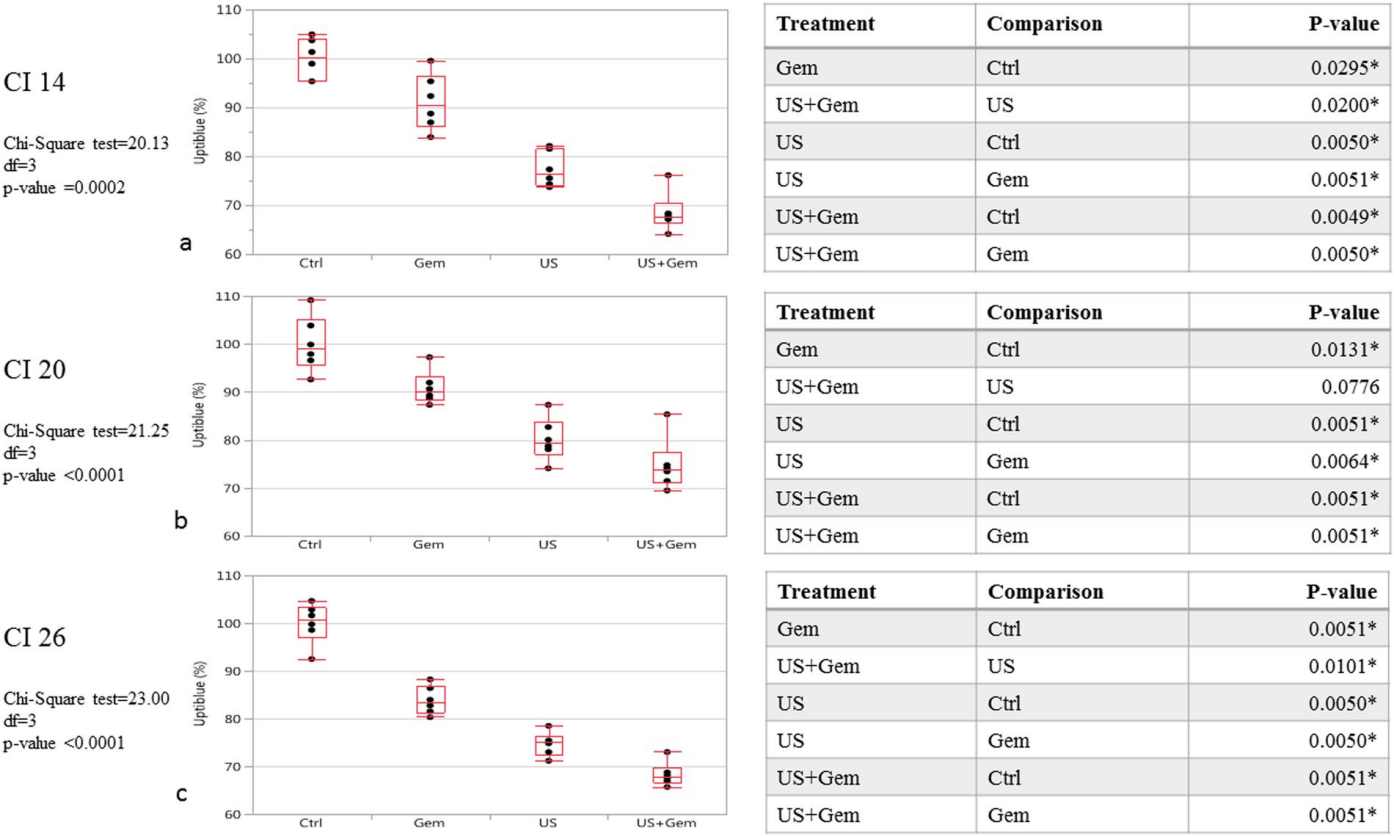
KPCF viability after various treatment conditions: Gem/US/US + Gem. (**a**) KPCF viability after US CI 14 treatment combined or not to Gem exposure. N = 6 spheroids for each condition. (**b**) KPCF viability after US CI 20 treatment combined or not to Gem exposure. N = 6 spheroids for each condition. (**c**) KPCF viability after US CI 26 treatment combined or not to Gem exposure. N = 6 spheroids for each condition.

Exposure of KPCF spheroids to 5 μM Gem or ultrasound cavitation at different cavitation regimens (CI or Cavitation Index) resulted in the following viability ratios: (a) 5 μM Gem alone *vs* CI 14 + 5 μM Gem: 91.2% (SD = 5.7) *vs* 68.6%, respectively (SD = 4.0) (p = 0.0050) (Fig. 4a); (b) 5 μM Gem alone *vs* CI 20 + 5 μM Gem: 90.8% (SD = 3.5) *vs* 74.7% (SD = 5.5) (p = 0.0051) (Fig. 4b); (c) 5 μM Gem alone vs CI 26 + 5 μM Gem: 83.8% (SD = 2.9) *vs* 68.3% (SD = 2.5) (p = 0.0051) (Fig. 4c). Measurements in each group were compared to control groups broken down to 100% viability.

### Viability after US treatment combined with Gem exposure on dissociated KPCF spheroids

**Figure 5:**
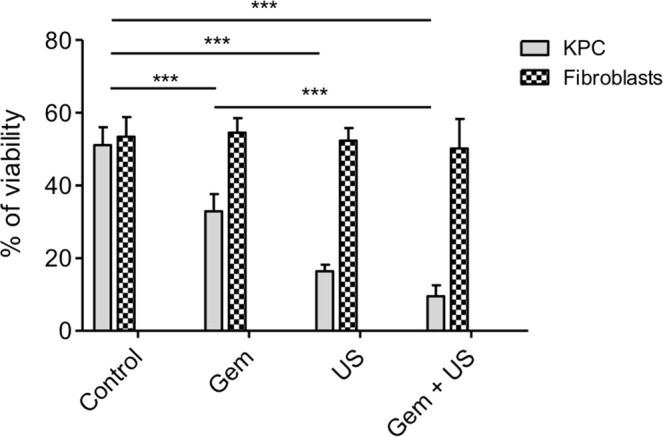
KPCF spheroid viability after US CI 20 treatment and Gem exposure. Cytometer analysis. Analysis based on 4 repeat experiments with 6 spheroids pooled for each condition.

**Figure 6:**
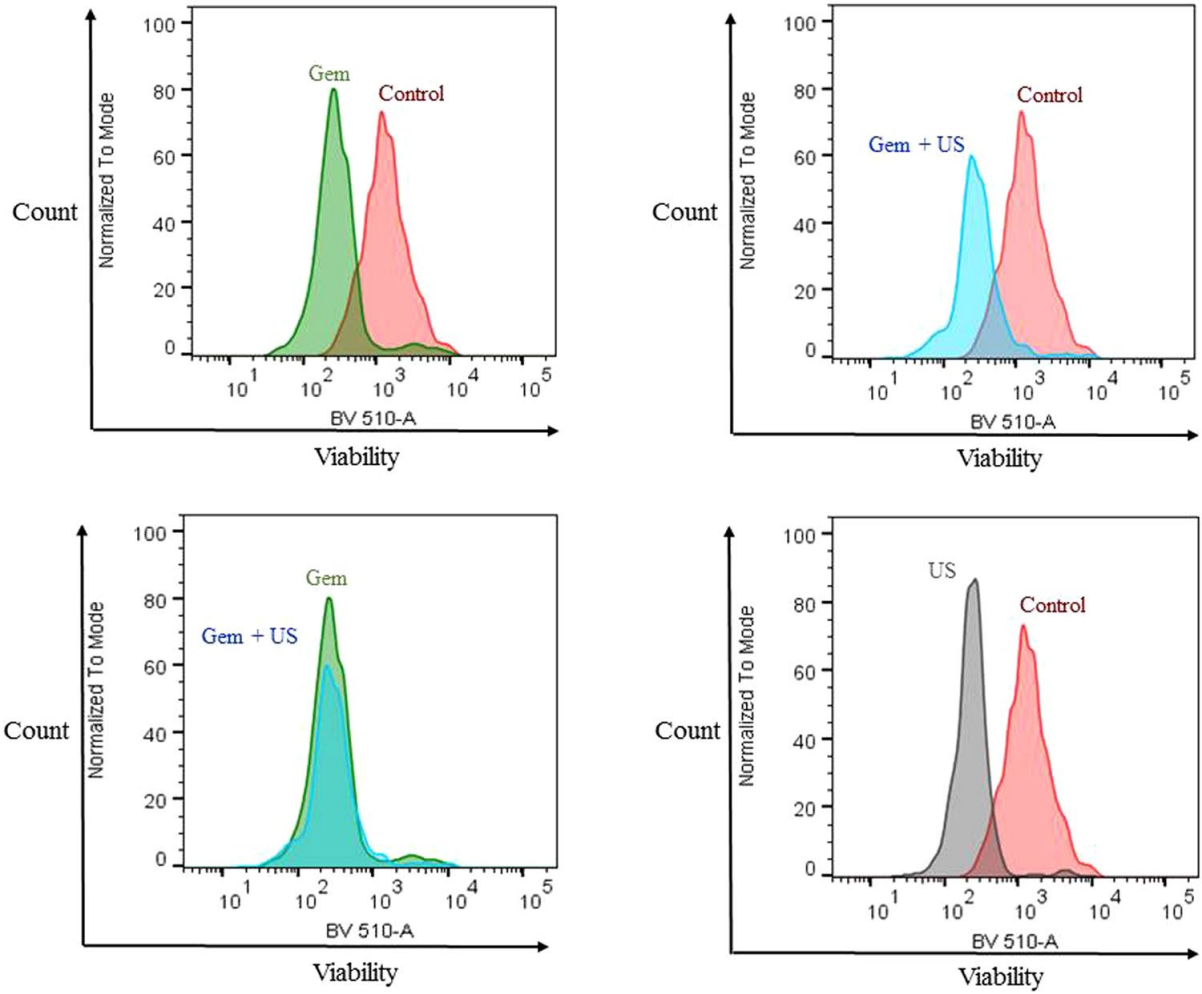
Example of the cytometer analysis assessing the viability measured by BV 510-A of KPC cells from dissociated KPCF spheroids after various treatments.

Post-treatment viability assays after spheroid trypsinization using cytometer analysis illustrated that only KPC cells were affected by the various treatments (Fig. 5 and example Fig. 6). At day 3 of growth, 51.1% (SD = 4.9) of KPC cells and 53.4% (SD = 5.4) of fibroblasts were still viable in the control group. Exposure to 5 μM Gem or US CI 20 induced a significant reduction of KPC viability (p = 0.028) but did not impact fibroblasts (p = 0.885). KPC cells submitted to US CI 20 cavitation only yielded 32.9% viability (SD = 4.7) compared to control group (p = 0.028); Gemcitabine yielded 16.4% viability (SD = 1.8) compared to control group (p = 0.028); US CI 20 + Gemcitabine yielded 9.5% viability (SD = 3) compared to control group (p = 0.028). Meanwhile, the viability of iMEF was not significantly affected.

## Discussion

In this study, we developed a KPCF 3D coculture model that offers a miniature representation of PDAC tumors and displays relevant features of the malignant microenvironment. The model was found to be highly reproducible and easy to handle, and lent itself to experiments with physical agents such as HIFU and US cavitation. The model has some inherent limitations, as evidenced by the cytometer analysis showing that after 3 days of growth, almost 50% of PDAC cells in KPCF spheroids died spontaneously. This observation can be explained by the large size of the spheroids at day 3 with a mean of 300 µm (SD = 2.4) at the broadest cross-section and the inherent characteristics of avascular *in vitro* spheroid. Besides the absence of vascularization, the physical properties of the KPCF spheroid may be different from those of a PDAC tumor and the absence of other important cellular populations, in particular those composing the inflammatory infiltrate and tumor lymphocytes, is another limitation of such a model. However, stromal cells supporting the development of PDAC in humans are recognized to be essentially cancer-associated fibroblasts deriving from mesenchymal stem cells or pancreatic stellate cells, which produce collagen and hyaluronan-based ECM and bear various specific surface proteins (a-SMA, FSP-1, FAP-a, PDGFR-b, SPARC…), whose activation/suppression can lead to either tumor suppression or promotion, particularly through the modulation of ECM stiffness^13–15^. It has also been suggested that a high interstitial pressure within the stroma resulting from a relatively immobile gel-fluid phase induces vascular collapse and hypoperfusion as a primary mechanism of treatment resistance in pancreatic cancer^16^. Our model differs from real PDAC in that it lacks any vascularization and intercellular trafficking is certainly much less complex than in the clinical setting. However, it possesses at least 3 crucial features of PDAC which make it a realistic approximation at the supracellular level: the presence of activated fibroblasts, the production of ECM and supposedly high interstitial pressure due to the avascular structure and the dense intercellular arrangement.

Our experiments show that Gemcitabine at the low dose of 5 μΜ was significantly less cytotoxic in the presence of iMEFs plus KPC cells compared with KPC cells alone, as shown by cell viability assays. Thus, the presence of activated fibroblasts or myofibroblasts in the KPCF model seems to play its role as a surrogate for stromal-related chemoresistance. US-induced cavitation associated with Gemcitabine enhanced a significant reduction of viability compared to Gemcitabine alone in the model, which supports our hypothesis that cavitation can help overcome stromal-related chemoresistance. Substantially, and although conditions were not exactly the same between experiments on the differential viability of KPC and KPCF spheroids under gemcitabine and cavitation experiments, it is suggested that cavitation helps significantly in overcoming the loss of efficacy of gemcitabine when iMEF are present in the spheroid. Moreover, Gem and US treatments reduced only the viability of KPC cells within KPCF spheroids and not that of fibroblasts, which is in line with an expected toxicity on malignant cells to be much higher than on cells belonging to the microenvironment. However, increments in cavitation indexes corresponding to increased US intensities (CI 14/20/26) did not seem to impact spheroid viability. Indeed, the mean values of KPCF viability after US CI 14/20/26 plus Gem were 68.6%, 74.7% and 68.3%, respectively but a statistically pooled comparison would not have been correct as values did not follow exactly the same distribution. The reasons for this kind of “on-off” effect of cavitation, would it be confirmed by further experiments, remains to be explained, since one might have expected a dose-effect correlation. A finer insight in the threshold for inertial cavitation triggering will be useful, and non-linear effects and possible annihilation of cell membrane damage by turbulence and undesirable spheroid motion within the assay tube (despite the thin and deep gel-moulded slot) still require further investigation. However, our findings support our main hypothesis of the ability of inertial cavitation to disrupt the non-malignant component of the spheroid (ie fibroblasts and ECM) and subsequently to enhance drug cytotoxicity.

Regarding the mechanisms of cavitation-induced biological damage in our model as well as in live tissues, different properties of inertial cavitation may be suggested: an increased cell membrane permeability, a disruption of the cell membrane increasing drug intake through sonoporation or ultrasound-specific thermal effects. Moreover, considering the fact that US application alone was able to decrease KPCF spheroid viability, mechanisms other than sonoporation are likely to occur. Among others, the synthesis of reactive hydroxyl species (ROS) could also be involved, as pyrolysis of the water vapor inside the microbubbles induced by inertial cavitation is known to generate ROS^17^. Although it remains difficult to understand to what extent the effectiveness of US-induced cavitation results from the specific effects of sonoporation, free radical production, ultrasound-specific thermal effects or from a particular combination of all of those, it is important to note that the rationale for the application of ultrasound herein described is very different from previously reported methods. In a widely described method, microbubbles of 1–10 μm in diameter can be supplied to the acoustic field in the form of contrast agents or sonosensitive nanocarriers and serve as nuclei for the inception of cavitation when submitted to moderate negative pressures (*eg* −0.1 MPa within a 1 MHz ultrasonic field). But cavitation can also be triggered without an external supply of bubbles, by applying negative pressures of much higher amplitude and longer pulses (*eg* up to −3.5 MPa and 2.5 ms, respectively, in our study). In that case, the higher the gas concentration within the medium, the easiest the inception of cavitation. The resulting microbubbles are formed of gas dissolved in tissue and water vapour^18–20^. For example, sonoporation as a method to enhance drug delivery generally requires lower US intensities, but is unlikely to exert any significant alteration of the extracellular matrix^21^, and the use of microbubbles or alternative bubble-generating particles to enhance cavitation relies on the presence of a neovascular network to convey the bubbles closer to the tumor structures, whose density is generally very low or absent in PDAC^22,23^. Intriguing clinical results were recently reported in PDAC patients treated with a combination of gemcitabine and commercially available microbubble-based contrast agents excited by low-intensity diagnostic US, with 5 out of 10 patients showing a decrease in tumor size and an unusually long median survival of 17.6 months compared to 8.9 months in historical controls^24^. However, the small size and methodological limitations of this uncontrolled study fail to convince that intravenously injected microbubbles can trigger cavitation and enhanced chemocytotoxicity at the right place and at the right moment. By using relatively high US intensities (although lower than in clinical extracorporeal lithotripsy) and a focused geometry of the apparatus allowing inertial cavitation generation with no need for an external provision of bubbles and regardless of the density of vessels, we demonstrate that US can disrupt the tumor microenvironment and enhance drug delivery in a manner that is likely more suitable to PDAC treatment than other methods.

To our knowledge, this study is the first evaluation of the effectiveness of US-induced cavitation associated with chemotherapy in a 3D coculture spheroid model of PDAC mimicking the tumor microenvironment which plays such an important role in chemoresistance. During PDAC development, activated PSCs are responsible for an imbalance between extra-cellular matrix (ECM) production and degradation that results in the production of a strong fibrotic stroma^25^. In the last decade, numerous *in vitro* 3D models of cancer have been developed to investigate the tumor behaviour and the relationship with its microenvironment^26,27^. The spheroid 3D architecture is able to mimic the ECM organization and hypoxic tumor regions^28^. Many protocols have been described for spheroid culture that often involve extensive fabrication procedure and time-consuming analysis^6^. The magnetic 3D cell levitation culture has revolutionized spheroid models by allowing an expeditious production of robust and relevant spheroids^29–31^. The PDAC coculture spheroid model appeared to be relevant for the evaluation of new pathways and innovative therapy as it can to some extent simulate both microenvironment and chemoresistance. The model ensured excellent reproducibility and easy and safe manipulations with no specimen loss or destruction. Still, a potential limitation has to be mentioned regarding the possible toxicity of nanoshuttle culture. Souza GR *et al*.^31^ have demonstrated that magnetic nanoparticles do not affect cell proliferation and metabolism. Moreover, our previous work has shown that nanoshuttle impacted neither cell viability, proliferation, or cell chemosensitivity under gemcitabine exposure^12^. In this study, previous monolayer fibroblast cultures had shown a decrease in viability of 14% (p = 0.0014) in case of fibroblasts cultured with nanoshuttle *vs* without nanoshuttle (not presented in this work).

Another caveat regards collagen, the presence of which has been demonstrated at day 10 of spheroid growth by the histological analysis. The presence of iron oxide within the nanoshuttle precluded an earlier histological assessment, at the time of US and chemotherapy exposure, and required to delay the histological analysis. It is therefore uncertain whether collagen and ECM production were detectable after only 3 days of growth. Finally, US application and Gem were only administered once. Considering successive treatments could have increased the synergistic efficacy on spheroids viability.

## Conclusion

Notwithstanding its inherent limitations, the KPCF 3D coculture model used in this study offered a credible and reproducible approximation of PDAC for *in vitro* studies, taking into account key elements of its microenvironment. Ultrasound-induced inertial cavitation associated to Gemcitabine was associated with a significant decrease in cell viability compared to Gemcitabine alone and has been determinant in overcoming the barrier against chemosensitivity created by iMEF.

## Methods

### Cell lines & Formation of coculture tumor spheroids

The KPC (Kras(G12D); p53(R172H); Pdx1-Cre) model of PDAC is a genetically engineered mouse model that incorporates the conditional expression of both mutant KrasG12D and p53R172H alleles in pancreatic cells^32^. KPC cell lines were cultured in Dulbecco’s Modified Eagle’s Medium (DMEM) with 10% Fetal Bovine Serum, supplemented with penicillin 1%, ciprofloxacin 1% and cultured in 5% CO_2_ at 37 °C.

**Figure 7:**
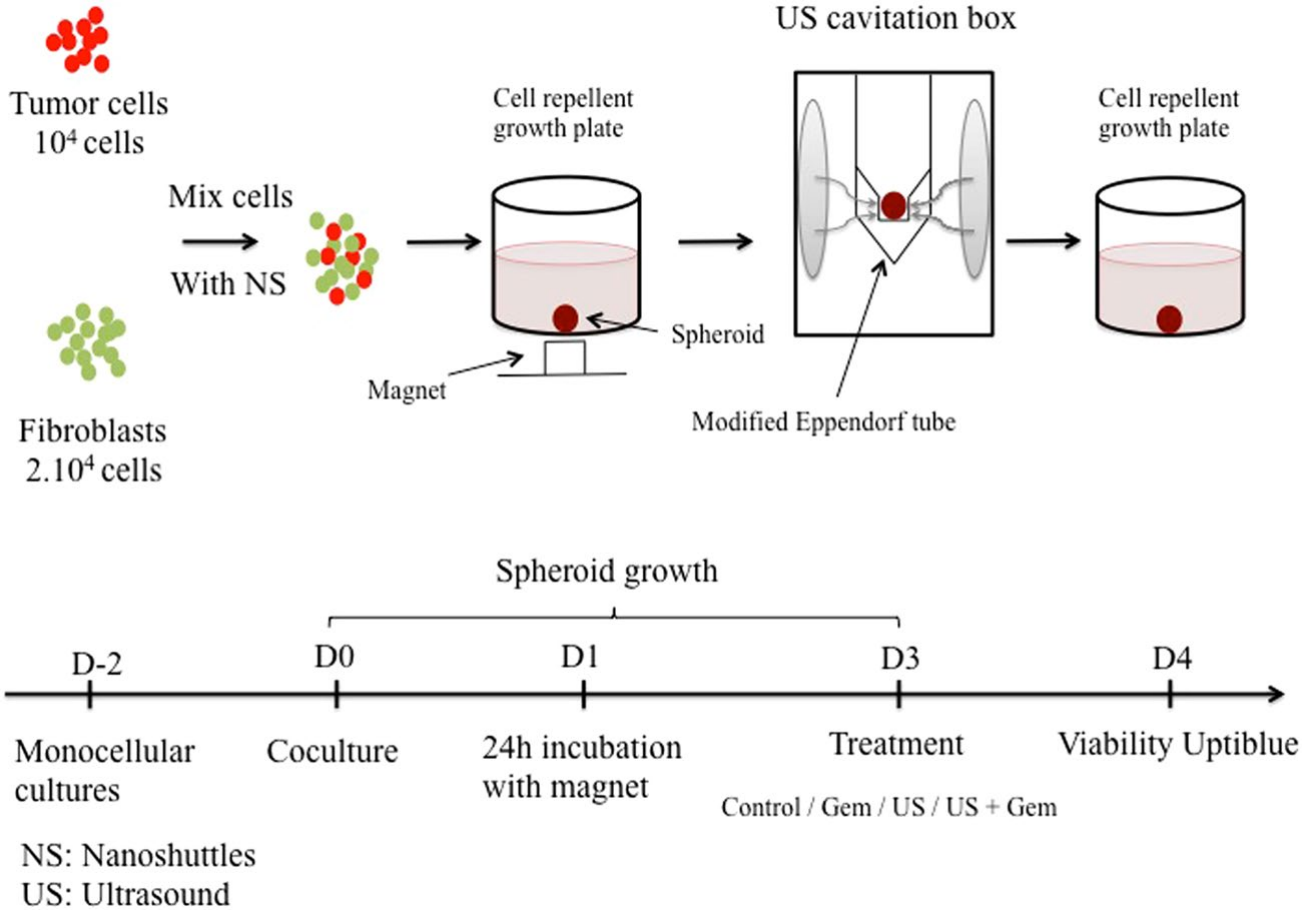
Coculture protocol/Formation of tumor spheroids/Treatment protocol design.

Murine embryonic fibroblasts (iMEF) isolated and immortalized by transduction with shp53 were cultured in the same conditions as KPC cells. KPC and iMEFs were both obtained by gracious donation from INSERM U1037 (Oncopole of Toulouse, France). KPC and iMEFs respectively expressed mCherry (red) and Green Fluorescent Protein (GFP) for clear immuno-fluorescent demarcation of both components. All experiments were done in accordance with french guidelines and regulations and approved by the Cochin Institute Scientific Committee. Spheroids were created by using a magnetic levitation technique as previously described by Noel P. *et al*.^29^. This magnetic 3D bioprinting protocol allowed rapid and reproducible production of spheroids and facilitated handling during subsequent manipulations and US exposures. Nanoshuttles (NS) composed of poly L-lysine, iron oxide and gold nanoparticles were used in both cell lines to label pancreatic tumor cells and fibroblasts at a concentration of 0.15 mg/mL. In a previous study^12^ we have shown that NS impacted neither cell viability, proliferation, or chemosensitivity under gemcitabine exposure. Fibroblasts were pre-treated 24 hours before coculture with a solution of TGFβ (Tumor Growth Factor β) (PeproTech®, USA) at 50 ng/mL to acquire a myofibroblastic phenotype and promote the desmoplastic reaction^33,34^. KPC and iMEF cells were co-seeded at 10,000 cells/well and 20,000 cells/well, respectively and grew into 3D spheroids in 800 μL medium using 24-well plates with cell repellent surface (Greiner Bio-One®, Germany). Spheroids were left untouched during 3 days before any additional treatment (Fig. 7).

### Image acquisition and spheroid characterization

Fluorescence microscopy images from spheroids were acquired using a confocal microscope Spinning Disk Leica® DM6000 (Leica®, Germany). MetaMorph® software (Molecular Devices®, USA) and ImageJ® software (ImageJ® software, NIH, USA) were used, respectively, for the acquisition and the analysis of images.

Histological analysis was performed after spheroid fixation with 4% paraformaldehyde on 5 micron-thick paraffin sections. As previously described, intra-spheroidal nanoparticle clearance was near-complete after eight days of growth^30^. Histological analyses were subsequently done after 10 days of growth. Spheroid sections were stained with standard hematoxylin and eosin as well as with Masson’s Trichrome for the detection of collagen.

### Spheroid treatment

#### US treatment

**Figure 8:**
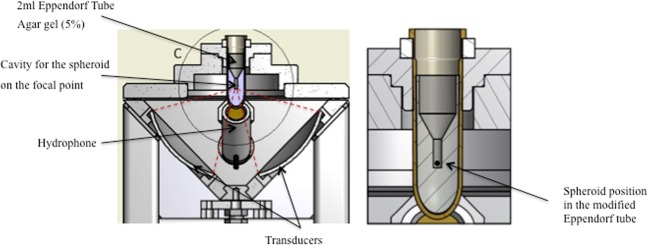
Spheroid position in the modified Eppendorf® tube.

A confocal US device similar to our previous study^12^ and also described by Chettab *et al*.^35^ was used and adapted for spheroids in order to deliver inertial cavitation in a controlled and reproducible manner (Fig. 8). The ultrasound delivery setup was composed of two piezoceramic focused transducers of 50 mm in diameter with a 50 mm curvature working at a frequency of F = 1.1 MHz, whose orthogonal arrangement allowed for precise beam focusing (focal volume 2 × 2 × 2 mm^3^). A waveform generator (PXI 5412 National Instruments, Austin, TX) created the excitatory signal and a 200 W power amplifier (LA200H, Kalmus, Bothell, WA) was used to amplify the signal, creating a burst of 2750 sinusoidal cycles with a PRF of 100 Hz (repetition period 10 ms) and a duty cycle of 25% (pulse duration 2.5 ms), thus generating essentially an inertial cavitation regimen. The peak negative pressure at the focal point was allowed to vary within a range of 0.4 to 3.5 MPa inside the Eppendorf tube (related acoustic intensities range 5–408 W.cm^−2^, decreasing to 1.3–102 W.cm^−2^ at a distance of 1 mm from the focal point). The transducers were immersed in the cavitation-inhibiting fluid Ablasonic™ (EDAP-TMS, France), thus preventing acoustic cavitation occurrence outside the sample submitted to ultrasound. An in-house hydrophone placed in the water tank recorded acoustic cavitation emissions from the focal area. A single spheroid was gently grabbed and transferred into a modified Eppendorf tube (Eppendorf®, Germany) designed for focused US so that the spheroid remained within the focal point throughout the procedure. Controlled cavitation was applied onto each single spheroid during 20 seconds. Different samples were exposed to incremental cavitation indexes (CI), namely CI 14, CI 20 and CI 26, corresponding to increasing ultrasound intensities.

### Chemotherapy exposure

Chemotherapy consisted in incubating a single spheroid with gemcitabine (Gem) (Gemcitabine Mylan, Mylan Medical, Paris, France, 40 mg/ml) with 100 mM stock solutions in dimethyl sulfoxide. Gem was administered in the culture medium at a concentration of 5 μM for 4 h at 37 °C.

### Experiments

A first experience aimed to evaluate how fibroblasts could influence the response to chemotherapy in spheroid microtumors. Multicellular spheroids from KPC and iMEFs co-culture, named KPCF spheroids, were compared to monotypic spheroids from KPC cell culture alone, named KPC spheroids. KPCF spheroids were composed of 10^4^ KPC cells and 2.10^4^ iMEFs per well whereas KPC spheroids were composed only of 10^4^ KPC cells per well. At day 3 of growth, six KPCF and KPC spheroids were treated with Gem and compared to six untreated KPCF and KPC spheroids serving as controls. For US experiments, individual spheroids were transferred from their culture plate into a modified Eppendorf® tube for US application. Spheroids were then returned to their initial media and incubated during 24 hours. All experiments were done in triplicate.

### Evaluation of spheroid viability

The reduction of Resazurin by metabolically active cells was the method used to assess viability (measured with 10% solution of Uptiblue®). Fluorescent intensity was then measured after 24 hours with spectrofluorimetry (Fusion®, Packard Bioscience, USA) using 530–560 nm excitation wavelength and 590 nm emission wavelength. All experiments were done in triplicate. Flow cytometric analysis for the viability assay was performed independently for both types of cells on a Fortessa® flow cytometer (BD®, Becton, Dickinson and Company, USA). The US CI 20 was selected for cytometer experiments of US-treated spheroids: after treatment, six spheroids were collected in an assay tube and dissociated using 1 ml trypsin solution (0,25% Trysin-EDTA, Gibco®, Thermofisher scientific). Cells were then marked with the BV-510 viability marker. FSC and SSC, providing green and red fluorescence signals at 530 and 650 nm, respectively, and BV-510 marker were evaluated for each cell. A total of 10,000 events were recorded for each tube. All experiments were performed four times with six dissociated KPCF spheroids per condition. Measurements were done under fixed instrument settings. Results were analyzed using Flowjo® software (LLC, USA).

### Statistical analysis

For statistical analysis, an ANOVA test was performed considering the number of conditions studied (more than 2). A Mann-Whitney test was used for cytometry results. Statistical analyses were performed on Graphpad Prism® software (GraphPad Software, Inc., La Jolla, California, USA). Results were considered to be statistically significant for a p value < 0.05.
